# Optimisation of novel 4, 8-disubstituted dihydropyrimido[5,4-*b*][1,4]oxazine derivatives as potent GPR 119 agonists

**DOI:** 10.1080/14756366.2019.1681988

**Published:** 2019-10-28

**Authors:** Yuanying Fang, Shaokun Zhang, Min Li, Lijuan Xiong, Liangxing Tu, Saisai Xie, Yi Jin, Yanhua Liu, Zunhua Yang, Ronghua Liu

**Affiliations:** a College of Pharmacy, Jiangxi University of Traditional Chinese Medicine, Nanchang, China; b National Engineering Research Center for Manufacturing Technology of TCM Solid Preparation, Jiangxi University of Traditional Chinese Medicine, Nanchang, China

**Keywords:** Optimisation, pyrimidodihydrooxazine, GPR 119 agonists, type 2 diabetes mellitus

## Abstract

GPR119 is a promising target for discovery of anti-type 2 diabetes mellitus agents. We described the optimisation of a novel series of pyrimido[5,4-*b*][1,4]oxazine derivatives as GPR119 agonists. Most designed compounds exhibited good agonistic activities. Among them, compound **10** and **15** demonstrated the potent EC_50_ values (13 and 12 nM, respectively) and strong inherent activities. Moreover, significant hypoglycaemic effect of compound **15** was observed by reducing the blood glucose AUC_0–2h_ at the dose of 30 mg/kg, which is stronger than Vildagliptin (23.4% reduction vs. 17.9% reduction).

## 1. Introduction

Type 2 diabetes mellitus (T2DM) is a complex chronic disease characterised by metabolic disorder and hyperglycaemia due to insulin resistance, hepatic glucose overproduction and/or insufficient insulin secretion^1–3^. Although there are a number of pharmacotherapy options for T2DM, most of current anti-diabetes drug have shown known adverse effects and loss of their overall efficacy in a long-term glycaemic control^4–6^. Thus, there is still a critical need of novel therapeutic targets or approaches for treatment T2DM by good glycaemic control.

G protein-coupled receptor 119 (GPR119) is a member of class A (rhodopsin-type) GPCR family, with highly expressed in pancreatic β-cells and the K and L cells of the gastrointestinal tract^7–9^. Activation of GPR119 increases the intracellular cyclic AMP (cAMP) level, which in turn directly stimulate the glucose-dependent insulin secretion and regulate glucagon-like peptide 1 (GLP-1), leading to improve the glucose tolerance in T2DM patients^10–14^. In addition, GPR119 agonists showed β-cells function preservation, which is also an important role in current T2DM therapy^15–17^. As a result, GPR119 agonists are used for discovery of anti-T2DM agents by lowering the blood glucose level and improving β-cells function. Indeed, numerous synthetic, small molecule GPR119 agonists were revealed by academia and industry to date, and some of which have advanced into clinical trials such as **MBX-2982**, **BMS-903452**, **LEZ763**, **ZYG-19**^18–30^. Despite tremendous endeavours, none of GPR119 agonists were approved to market by FDA up to now.

**Figure 1.**
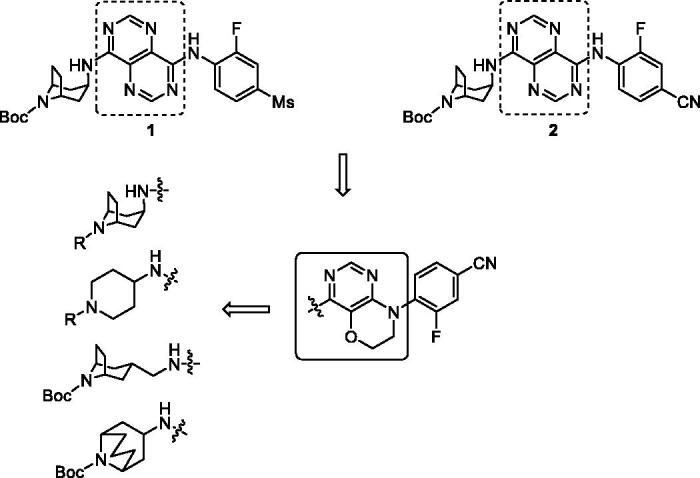
The design of target compounds.

In our efforts to discover GPR119 agonists, we previously have evaluated some series of pyrimidine derivatives, and some compounds displayed quite good agonistic potency^31^^,^^32^. Among them, pyrimidopyrimidine compounds **1** and **2** exhibited single digit EC_50_ values (2.2 nM and 8.1 nM respectively); however, these two agonists did not show the significant glucose-lowering effect in oral glucose tolerance test (oGTT) in mice compared with positive control. Therefore, we sequentially attempted to optimise the core fragment with the aim to enhance the biological activity both *in vitro* and *in vivo*. With this purpose, we introduced pyrimido[5,4-*b*][1,4]oxazine as the core using the strategy of scaffold hopping. We also identified whether introduction of several conformation restricted azabicyclic amines were beneficial to agonistic potency or not (Figure 1). In this paper, we described our optimisation to synthesise and evaluate a series of novel pyrimido[5,4-*b*][1,4]oxazine derivatives as GPR119 agonists, also including an *in vivo* efficacy study.

## 2. Results and discussion

### 2.1. Chemistry

**Scheme 1.**
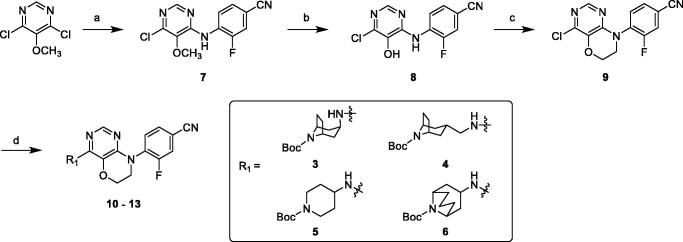
Synthesis of compounds **10**–**13**. Reagents and conditions: (a) 4-amino-3-fluorobenzonitrile, K_2_CO_3_, DMF, 65 °C, overnight. (b) 1 M BBr_3_ in DCM, anhydrous DCM, r. t. – reflux, 2 h. (c) 1-bromo-2-chloroethane, K_2_CO_3_, DMF, 40 °C, overnight. (d) amines **3**–**6**, Pd_2_(dba)_3_, X-Phos, Cs_2_CO_3_, 1,4-Dioxane, reflux, under N_2_ overnight.

The intermediates amine or bicyclic amines **3**–**6** could be purchased or synthesised according to the reported procedures^33–36^. Coupling reaction of 4,6-dichloro-5-methoxypyrimidine with 4-amino-3-fluorobenzonitrile in DMF under basic condition afforded compound **7**, followed by demethylation using BBr_3_ solution in dichloromethane under reflux condition gave hydroxyl compound **8**. Cyclisation of **8** with 1-bromo-2-chloroethane and K_2_CO_3_ in DMF generated key intermediate **9**. Buchwald–Hartwig reaction of **9** and amines **3**–**6** with Pd_2_(dba)_3_, X-Phos and Cs_2_CO_3_ under reflux conditions and N_2_ atmosphere overnight resulted in target compounds **10**–**13** (Scheme 1).

**Scheme 2.**
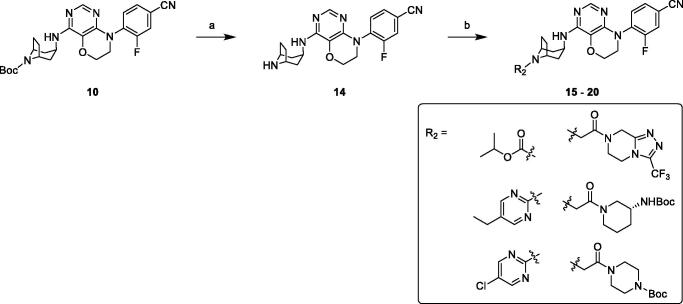
Synthesis of compounds **15**–**20**. Reagents and conditions: (a) 3 M HCl in EtOH, r. t., overnight. (b) chloro-fragments, Et_3_N, DCM, r. t., overnight or Cs_2_CO_3_, DMF, r. t., overnight.

Removing Boc group of derivative **10** in 3 M HCl ethanol solution obtained amine compound **14** in good yield, which was treated with various chloro-fragments in base conditions to receive desired final compounds **15**–**20** (Scheme 2).

**Scheme 3.**
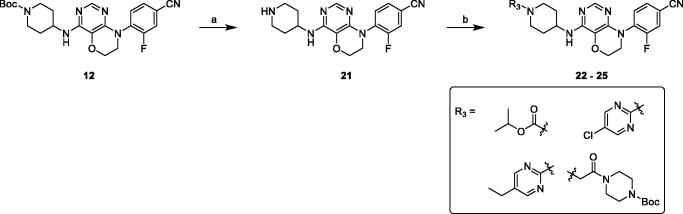
Synthesis of compounds **22**–**25**. Reagents and conditions: (a) 3 M HCl in EtOH, r. t., overnight. (b) chloro-fragments, Et_3_N, DCM, r. t., overnight or Cs_2_CO_3_, DMF, r. t., overnight.

The general synthetic procedures of the pyrimidooxazine derivatives **22**–**25** were synthesised as shown in Scheme 3, following similar methods with compounds **15**–**20**.

### 2.2. Biological activity

#### 2.2.1. GPR119 activation

GPR119 agonistic activity of target compounds **10**–**13**, **15**–**20**, and **22**–**25** were measured using a reporter assay with the human GPR119 receptor stably expressed in CHO K1 cells. A GPR119 agonist **GSK-1292263** was chose for the reference. The results expressed the activity as EC_50_ values and the inherent activity (IA) as percentages (%max) of response which were compared to the reference **GSK-1292263** (defined the maximal effect activation).

At first, we evaluated pyrimidooxazine derivatives **10**–**13** for the GPR119 agonistic activity and intrinsic activity (Table 1). As a result, good activities were observed with compounds **10** and **12** (EC_50_ = 13 and 200 nM respectively), which contained tropine amine and piperidine amine scaffolds with moderate lipophilicity (Clog*P* = 4.0 and 3.5 respectively).

**Table 1.** GPR119 agonistic activities of compounds **10**–**13**.

Compound	Structure	GPR119 activation		Clog*P*^b^
		EC_50_ (nM)	%max^a^	
**10**		13	83.9	4.0
**11**		1800	45.9	4.6
**12**		200	63.5	3.5
**13**		>10000	22.7	4.5
**GSK-1292263**		6.6	100	

^a^ %max: cAMP stimulation % compared to maximal effect of **GSK1292263**.

^b^ Clog*P* was calculated using ACD software from Discovery Studio 4.5.

Next, we focussed on the modification of Boc group in head part with various moieties. The biological results were shown in Table 2, all compounds exhibited moderate to potent agonistic activities (EC_50_ values range from 250 nM to 12 nM). The substitution of 2-pyrimidyl on the nitrogen of tropine ring yielded compounds **16** and **17**, which showed the moderate EC_50_ values (130 nM and 250 nM, respectively). However, the compounds **23** and **24**, bearing same substituents on the nitrogen of piperidine ring, demonstrated significant agonistic activities (EC_50_ = 44 nM and 40 nM) and lower lipophilicity. But the reverse results were observed for carbamate substituted derivatives (**15** vs. **22**), and compound **15** exhibited 10 times EC_50_ values than compound **22**. Furthermore, compound **15** revealed the strongest inherent activity (%max 146.5%) and best agonistic activity (EC_50_ = 12 nM) with suitable Clog*P* value (3.8). Consequently, compounds **15** and **10** were selected and examined the oral glucose tolerance test (oGTT) *in vivo* as promising GPR119 agonists.

**Table 2.** GPR119 agonistic activities of compounds **15–20** and **22–25**.

Compound	Structure	GPR119 activation		Clog*P*^b^
		EC_50_ (nM)	%max^a^	
**15**		12	146.3	3.8
**16**		130	60.5	4.6
**17**		250	71.2	4.3
**18**		110	100	3.0
**19**		68	75.6	3.7
**20**		82	79.6	3.3
**22**		120	66.1	3.3
**23**		44	70.7	4.0
**24**		40	62.7	3.8
**25**		90	100	2.7
**GSK-1292263**		6.6	100	

^a^ %max: cAMP stimulation % compared to maximal effect of **GSK1292263**.

^b^ Clog*P* was calculated using ACD software from Discovery Studio 4.5.

#### 2.2.2. oGTT in mice

**Figure 2.**
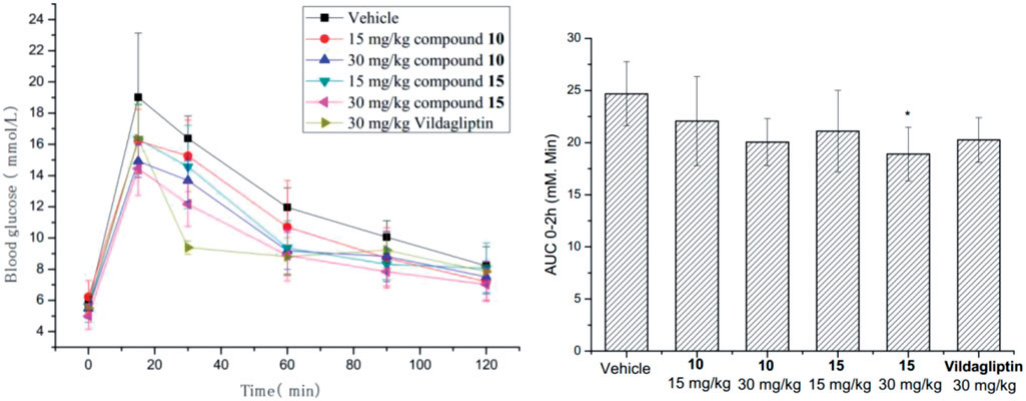
Single dose of compounds **10** and **15** on oGTT in C57BL/6N mice. The results are presented as the mean ± SE. *p < 0.05 compared to vehicle group (*n* = 8).

Based on the good agonistic activity, we conducted oGTT of compounds **10** and **15** with a single dose (15 and 30 mg/kg) in C57BL/6N mice with DPP-4 inhibitor vildagliptin as a positive control. The results were outlined in Figure 2, compounds **10** and **15** both showed blood glucose reduction effect in dose-dependent manner. Vildagliptin reduced the area under curve from 0 to 120 min (AUC_0–120 min_) by 17.9% (Vehicle: 24.69 ± 3.08, Vildagliptin: 20.26 ± 2.14) at the dose of 30 mg/kg. Meanwhile compounds **10** and **15** reduced AUC_0–120 min_ by 10.7% (22.07 ± 4.28) and 14.5% (21.10 ± 3.92) at the dosage of 15 mg/kg, respectively. However, **10** and **15** showed significant improved efficacy by the reduction of value to 18.8% (20.05 ± 2.27) and 23.4% (18.91 ± 2.58) at the dosage of 30 mg/kg, respectively.

## 3. Conclusion

In summary, we have designed, synthesised and biologically evaluated a series of novel pyrimido[5,4-*b*][1,4]oxazine derivatives as potent GPR119 agonists. *In vitro*, half derivatives exhibited strong EC_50_ values (<100 nM). Among the aliphatic amine moieties of this scaffold, the compound **10** with tropine amine ring displayed much more potent agonistic activity than piperidine amine and other rigid bicyclic amines. In the further optimisation of *N*-substitution, only isopropyl carbamate of tropine ring **15** improved the EC_50_ values and showed the greatest inherent activity. Accordingly, compounds **10** and **15** were conducted the oGTT in C57BL/6N mice. Both two agonists demonstrated blood glucose reduction effect in a dose-dependent manner. Furthermore, the optimised compound **15** was exerted improved 23.4% reduction in blood glucose AUC_0–2h_ at the dose of 30 mg/kg comparing with Vildagliptin (17.9% reduction). Follow-up studies and their results will be reported in due course.

## 4. Experimental

### 4.1. Chemistry

All starting materials were obtained from commercial suppliers and used without further purification. ^1^H-NMR and 13C-NMR spectra were recorded on a Bruker AVANCE III HD 600 (600 Hz) spectrometer. Chemical shifts are reported in parts per million (ppm) downfield relative to tetramethylsilane as an internal standard. Peak splitting patterns are abbreviated as s (singlet), br s (broad singlet), d (doublet), t (triplet), dd (doublet of doublet), and m (multiplet). MS spectra were recorded on a Thermo Fisher (LCQ Fleet). HR-MS spectra were recorded on an AB SCIEX (Triple TOF 5600+). TLC was performed on silica F254 purchased from Branch of Qingdao Haiyang Chemical Co. (Qingdao, China) and detected by UV light at 254, 365 nm or by charring with sulphuric acid. Column chromatography was performed on silica gel column (200–300 mesh, Branch of Qingdao Haiyang Chemical Co.). Analytical HPLC was performed on a Waters Acquity^®^ Arc™ with 2998 PDA detector and all final compounds possessed purities of >90% after purification.

#### 4.1.1. 4-((6-Chloro-5-methoxypyrimidin-4-yl)amino)-3-fluorobenzonitrile (7)

To a solution of 4,6-dichloro-5-methoxypyrimidine (1 g, 5.7 mmol) in DMF (20 ml), 4-amino-3-fluorobenzonitrile (0.6 g, 4.4 mmol) and K_2_CO_3_ (2.4 g, 17 mmol) were added. The reaction was stirrd at 65 °C for overnight. Then the mixture was poured into ice water. The mix solution was extracted with ethyl acetate for two times, washed with brine for two times. The organic layer was dried over MgSO_4_, filtered and evaporated. The residue was purified by column chromatography (petroleum ether: EtOAc = 3: 1) to afford the desired product as a claybank solid (0.68 g, 55%). ^1^H-NMR (600 MHz, CDCl_3_) δ (ppm): 8.94 (t, *J* = 8.8 Hz, 1H), 8.35 (s, 1H), 7.73 (s, 1H), 7.59 (d, *J* = 8.8 Hz, 1H), 7.52 (d, *J* = 10.2 Hz, 1H), 3.10 (s, 3H). MS-ESI: [M + H]^+^: 279.3.

#### 4.1.2. 4-((6-Chloro-5-hydroxypyrimidin-4-yl)amino)-3-fluorobenzonitrile (8)

To a solution of compound **7** (0.5 g, 1.9 mmol) in anhydrous dichloromethane (15 ml), 1 M BBr_3_ in dichloromethane solution (5.7 ml, 5.7 mmol) was added at room temperature (r. t.) The reaction was reflux for 2 h. Then the reaction was quenched by water. The mixture was extracted with dichloromethane for two times, washed by brine for two times. The organic layer was dried over MgSO_4_, filtered and evaporated. The residue was purified by column chromatography (petroleum ether: EtOAc = 1: 1) to give the desired product as a yellow solid (0.3 g, 72%). ^1^H-NMR (600 MHz, CDCl_3_) δ (ppm): 8.92 (t, *J* = 8.4 Hz, 1H), 8.32 (s, 1H), 7.68 (s, 1H), 7.53 (d, *J* = 8.6 Hz, 1H), 7.46 (d, *J* = 10.8 Hz, 1H). MS-ESI: [M + H]^+^: 265.1.

#### 4.1.3. 4-(4-Chloro-6,7-dihydro-8H-pyrimido[5,4-b][1,4]oxazin-8-yl)-3-fluorobenzonitrile (9)

To a solution of compound **8** (0.4 g, 1.4 mmol) in DMF (10 ml), 1-bromo-2-chloroethane (0.62 g, 4.3 mmol) and K_2_CO_3_ (0.6 g, 4.3 mmol) were added. The reaction was stirred at 40 °C for overnight. Then the mixture was poured into ice water. The mix solution was extracted with ethyl acetate for two times, washed with brine for two times. The organic layer was dried over MgSO_4_, filtered and evaporated. The residue was purified by column chromatography (petroleum ether: EtOAc = 2: 1) to afford the desired product as a claybank solid (0.68 g, 55%). ^1^H-NMR (600 MHz, CDCl_3_) δ (ppm): 8.04 (s, 1H), 8.35 (s, 1H), 7.73–7.44 (m, 3H), 4.51 (t, *J* = 4.4 Hz, 2H), 3.95 (t, *J* = 4.3 Hz, 2H). MS-ESI: [M + H]^+^: 291.5.

#### 4.1.4. General procedure of compounds 10–13

To the solution of compound **9** (0.22 mmol) and substituted amines (0.22 mmol) in 1,4-dioxane (2 ml), Pd_2_(dba)_3_ (0.05 mmol), X-Phos (0.05 mmol), and Cs_2_CO_3_ (0.55 mmol) were added. The reaction was heated to reflux under nitrogen gas for overnight. Then the mixture was diluted with ethyl acetate, washed with brine, dried over MgSO_4_, and evaporated. The residue was purified by column chromatography to give the product.

#### 4.1.5. tert-Butyl (endo)-3-((8-(4-cyano-2-fluorophenyl)-7,8-dihydro-6H-pyrimido[5,4-b][1,4]oxazin-4-yl)amino)-8-azabicyclo[3.2.1]octane-8-carboxylate (10)

Yellowish solid, 52% yield. ^1^H-NMR (600 MHz, CDCl_3_) δ (ppm): 7.93 (s, 1H), 7.54–7.50 (m, 1H), 7.47 (m, 2H), 5.38 (d, *J* = 7.0 Hz, 1H), 4.46–4.37 (m, 2H), 4.33 (m, 2H), 4.22 (s, 1H), 3.89 (s, 2H), 2.39 (s, 1H), 2.30–2.20 (m, 1H), 2.12 (t, *J* = 7.4 Hz, 2H), 2.07–1.92 (m, 2H), 1.84 (d, *J* = 27.1 Hz, 2H), 1.49 (s, 9H). 13C-NMR (150 MHz, CDCl_3_) δ (ppm): 156.4 (d, *J* = 250.5 Hz), 153.4, 151.3, 149.8, 143.9, 135.4 (d, *J* = 10.5 Hz), 128.5 (d, *J* = 3.5 Hz), 128.4 (d, *J* = 2.6 Hz), 121.8, 120.6 (d, *J* = 23.7 Hz), 117.6 (d, *J* = 2.6 Hz), 109.4 (d, *J* = 9.2 Hz), 79.5, 64.5, 53.1, 52.2, 48.2 (d, *J* = 3.9 Hz), 43.1, 35.8, 35.3, 28.5 (x3), 28.3, 27.9. HRMS-TOF (*m/z*) calcd for C_25_H_29_FN_6_O_3_ [M + H]^+^: 481.2358, found 481.2436.

#### 4.1.6. tert-Butyl (exo)-3-(((8-(4-cyano-2-fluorophenyl)-7,8-dihydro-6H-pyrimido[5,4-b][1,4]oxazin-4-yl)amino)methyl)-8-azabicyclo[3.2.1]octane-8-carboxylate (11)

Yellowish solid, 45% yield. ^1^H-NMR (600 MHz, CDCl_3_) δ (ppm): 7.92 (s, 1H), 7.55–7.51 (m, 1H), 7.50–7.44 (m, 2H), 5.00 (t, *J* = 6.1 Hz, 1H), 4.39–4.35 (m, 2H), 4.30 (s, 1H), 4.20 (s, 1H), 3.95–3.81 (m, 2H), 3.37 (d, *J* = 23.7 Hz, 2H), 2.29–2.14 (m, 1H), 1.97 (s, 2H), 1.72–1.63 (m, 4H), 1.65 (s, 2H), 1.49 (s, 9H). HRMS-TOF (*m/z*) calcd for C_26_H_31_FN_6_O_3_ [M + H]^+^: 495.2514, found 495.2592.

#### 4.1.7. tert-Butyl 4-((8-(4-cyano-2-fluorophenyl)-7,8-dihydro-6H-pyrimido[5,4-b][1,4]oxazin-4-yl)amino)piperidine-1-carboxylate (12)

Yellowish solid, 76% yield. ^1^H-NMR (600 MHz, CDCl_3_) δ (ppm): 7.93 (s, 1H), 7.56–7.51 (m, 1H), 7.48 (m, 2H), 4.83 (d, *J* = 8.1 Hz, 1H), 4.38 (t, *J* = 4.3 Hz, 2H), 4.17–4.11 (m, 3H), 3.89–3.87 (t, *J* = 3.7, 2H), 2.98 (m, 2H), 2.07–2.05 (m, 2H), 1.49 (s, 9H), 1.43–1.40 (m, 2H). 13C-NMR (150 MHz, CDCl_3_) δ (ppm): 156.4 (d, *J* = 250.7 Hz), 154.8, 151.2, 149.7, 144.2, 135.4 (d, *J* = 10.5 Hz), 128.5 (d, *J* = 3.6 Hz), 128.4 (d, *J* = 2.6 Hz), 121.7, 120.6 (d, *J* = 23.7 Hz), 117.6 (d, *J* = 2.7 Hz), 109.5 (d, *J* = 9.0 Hz), 79.7, 64.3, 48.2 (d, *J* = 3.8 Hz), 47.6, 43.0, 32.5 (x2), 28.5 (x3). HRMS-TOF (*m/z*) calcd for C_23_H_27_FN_6_O_3_ [M + H]^+^: 455.2201, found 455.2275.

#### 4.1.8. tert-Butyl 3-((8-(4-cyano-2-fluorophenyl)-7,8-dihydro-6H-pyrimido[5,4-b][1,4]oxazin-4-yl)amino)-9-azabicyclo[3.3.1]nonane-9-carboxylate (13)

Yellowish solid, 52% yield. ^1^H-NMR (600 MHz, CDCl_3_) δ (ppm): 7.91 (s, 1H), 7.52 (t, *J* = 7.8 Hz, 1H), 7.50–7.43 (m, 2H), 4.71 (d, *J* = 8.3 Hz, 1H), 4.60 (t, *J* = 9.0 Hz, 1H), 4.46 (d, *J* = 12.3 Hz, 1H), 4.36 (dt, *J* = 6.2, 4.8 Hz, 2H), 4.03 (m, 1H), 3.93–3.81 (m, 2H), 2.52 (m, 2H), 2.09–1.94 (m, 2H), 1.85 (m, 1H), 1.77–1.74 (m, 1H), 1.72–1.69 (m, 1H), 1.66-.1.61 (m, 1H), 1.58–1.57 (m, 1H), 1.50 (s, 9H), 1.43–1.41 (m, 2H). HRMS-TOF (*m/z*) calcd for C_26_H_31_FN_6_O_3_ [M + H]^+^: 495.2514, found 495.2600.

#### 4.1.9. 4-(4-(((endo)-8-Azabicyclo[3.2.1]octan-3-yl)amino)-6,7-dihydro-8H-pyrimido[5,4-b][1,4]oxazin-8-yl)-3-fluorobenzonitrile (14)

To a solution of compound **10** (1 mmol) in 3 M HCl/EtOH (40 ml) was stirred at r. t. for overnight. Then the mixture was filtered to obtain the product **14**, which was used for next step without purification. MS-ESI: [M + H]^+^: 381.3.

#### 4.1.10. Isopropyl (endo)-3-((8-(4-cyano-2-fluorophenyl)-7,8-dihydro-6H-pyrimido[5,4-b][1,4]oxazin-4-yl)amino)-8-azabicyclo[3.2.1]octane-8-carboxylate (15)

To a solution of compound **14** (0.1 g, 0.26 mmol) in dichloromethane (3 ml), isopropyl carbonochloridate (30 μL, 0.34 mmol) and Et_3_N (73 μL, 1 mmol) were added. The reaction was stirred at r.t. for overnight. Then mixture was diluted with dichloromethane, washed with brine for two times. The organic layer was dried over MgSO_4_, filtered and evaporated. The residue was purified by column chromatography (petroleum ether: EtOAc = 1: 1) to afford the desired product as a light yellow solid (70 mg, 58%). ^1^H-NMR (600 MHz, CDCl_3_) δ (ppm): 7.93 (s, 1H), 7.54–7.51 (m, 1H), 7.48 (m, 2H), 5.38 (d, *J* = 6.9 Hz, 1H), 4.98 (dt, *J* = 12.5, 6.2 Hz, 1H), 4.40–4.37 (m, 3H), 4.34 (m, 2H), 3.89 (m, 2H), 2.37 (m, 1H), 2.24 (m, 1H), 2.13 (m, 2H), 2.02 (m, 2H), 1.87 (m, 2H), 1.28 (s, 3H), 1.27 (s, 3H). 13C-NMR (150 MHz, CDCl_3_) δ (ppm): 156.4 (d, *J* = 250.5 Hz), 153.6, 151.3, 149.8, 143.9, 135.4 (d, *J* = 10.5 Hz), 128.5 (d, *J* = 3.6 Hz), 128.4 (d, *J* = 2.5 Hz), 121.8, 120.6 (d, *J* = 23.7 Hz), 117.6 (d, *J* = 2.4 Hz), 109.4 (d, *J* = 9.2 Hz), 68.1, 64.5, 53.1, 52.6, 48.2 (d, *J* = 4.4 Hz), 43.1, 35.9, 35.4, 28.3, 27.9, 22.3 (x2). HRMS-TOF (*m/z*) calcd for C_24_H_27_FN_6_O_3_ [M + H]^+^: 467.2201, found 467.2282.

#### 4.1.11. General procedure of compounds 16–20

To a solution of compound **14** (0.26 mmol) in DMF (3 ml), chloro-fragments (0.34 mmol) and K_2_CO_3_ (1 mmol) were added. The reaction was stirred at r. t. for overnight. Then the mixture was poured into ice water. The mix solution was extracted with ethyl acetate for two times, washed with brine for 2 times. The organic layer was dried over MgSO_4_, filtered and evaporated. The residue was purified by column chromatography to afford the desired product.

#### 4.1.12. 4-(4-(((endo)-8-(5-Ethylpyrimidin-2-yl)-8-azabicyclo[3.2.1]octan-3-yl)amino)-6,7-dihydro-8H-pyrimido[5,4-b][1,4]oxazin-8-yl)-3-fluorobenzonitrile (16)

Yellowish solid, 47% yield. ^1^H-NMR (600 MHz, CDCl_3_) δ (ppm): 8.22 (s, 2H), 7.92 (s, 1H), 7.55–7.51 (m, 1H), 7.47 (m, 2H), 5.52 (d, *J* = 7.2 Hz, 1H), 4.78 (s, 2H), 4.43–4.37 (m, 2H), 4.30 (q, *J* = 6.6 Hz, 1H), 3.92–3.85 (m, 2H), 2.50 (q, *J* = 7.6 Hz, 2H), 2.38 (m, 2H), 2.25–2.18 (m, 2H), 2.13 (m, 2H), 1.89 (d, *J* = 14.2 Hz, 2H), 1.23 (t, *J* = 7.6 Hz, 3H). 13C-NMR (150 MHz, CDCl_3_) δ (ppm): 159.1, 157.5 (x2), 156.4 (d, *J* = 250.7 Hz), 151.4, 149.9, 143.8, 135.5 (d, *J* = 10.4 Hz), 128.5 (d, *J* = 3.5 Hz), 128.4 (d, *J* = 2.6 Hz), 124.6, 121.8, 120.6 (d, *J* = 23.7 Hz), 117.6 (d, *J* = 2.7 Hz), 109.4 (d, *J* = 9.2 Hz), 64.5, 52.6 (x2), 48.3 (d, *J* = 3.9 Hz), 43.5, 34.7 (x2), 28.1 (x2), 22.8, 15.6. HRMS-TOF (*m/z*) calcd for C_26_H_27_FN_8_O [M + H]^+^: 487.2365, found 487.2462.

#### 4.1.13. 4-(4-(((endo)-8-(5-Chloropyrimidin-2-yl)-8-azabicyclo[3.2.1]octan-3-yl)amino)-6,7-dihydro-8H-pyrimido[5,4-b][1,4]oxazin-8-yl)-3-fluorobenzonitrile (17)

Yellowish solid, 51% yield. ^1^H-NMR (600 MHz, CDCl_3_) δ (ppm): 8.27 (s, 2H), 7.92 (s, 1H), 7.53 (t, *J* = 7.8 Hz, 1H), 7.51–7.44 (m, 2H), 5.48 (d, *J* = 7.1 Hz, 1H), 4.87–4.67 (m, 2H), 4.49–4.38 (m, 2H), 4.28 (q, *J* = 6.6 Hz, 1H), 3.95–3.83 (m, 2H), 2.34 (m, 2H), 2.26–2.19 (m, 2H), 2.14 (m, 2H), 1.96–1.88 (m, 2H). 13C-NMR (150 MHz, CDCl_3_) δ (ppm): 158.0, 156.4 (d, *J* = 250.6 Hz), 156.3 (x2), 151.3, 149.9, 143.9, 135.4 (d, *J* = 10.5 Hz), 128.5 (d, *J* = 3.6 Hz), 128.4 (d, *J* = 2.5 Hz), 121.8, 120.6 (d, *J* = 23.7 Hz), 118.1, 117.6 (d, *J* = 2.6 Hz), 109.4 (d, *J* = 9.2 Hz), 64.5, 52.8 (x2), 48.2 (d, *J* = 3.9 Hz), 43.4, 34.6 (x2), 28.1 (x2). HRMS-TOF (*m/z*) calcd for C_24_H_22_ClFN_8_O [M + H]^+^: 493.1662, found 493.1751.

#### 4.1.14. 3-Fluoro-4-(4-(((endo)-8-(2-oxo-2-(3-(trifluoromethyl)-5,6-dihydro-[1,2,4]triazolo[4,3-a]pyrazin-7(8H)-yl)ethyl)-8-azabicyclo[3.2.1]octan-3-yl)amino)-6,7-dihydro-8H-pyrimido[5,4-b][1,4]oxazin-8-yl)benzonitrile (18)

Yellowish solid, 44% yield. ^1^H-NMR (600 MHz, CDCl_3_) δ (ppm): 7.91 (s, 1H), 7.53–7.50 (m, 1H), 7.49–7.43 (m, 2H), 5.30 (m, 2H), 5.05 (s, 1H), 4.38 (m, 2H), 4.33–4.16 (m, 4H), 4.11 (t, *J* = 5.6 Hz, 1H), 3.87 (t, *J* = 4.3 Hz, 2H), 3.36 (d, *J* = 3.8 Hz, 2H), 3.26 (m, 2H), 2.29–2.08 (m, 4H), 1.97 (m, 2H), 1.82 (m, 2H). HRMS-TOF (*m/z*) calcd for C_28_H_28_FN_10_O_2_ [M + H]^+^: 613.2406, found 613.2508.

#### 4.1.15. tert-Butyl ((R)-1-(2-((endo)-3-((8-(4-cyano-2-fluorophenyl)-7,8-dihydro-6H-pyrimido[5,4-b][1,4]oxazin-4-yl)amino)-8-azabicyclo[3.2.1]octan-8-yl)acetyl)piperidin-3-yl)carbamate (19)

Yellowish solid, 50% yield. ^1^H-NMR (600 MHz, CDCl_3_) δ (ppm): 7.92 (s, 1H), 7.52 (m, 1H), 7.47 (m, 2H), 5.47–5.32 (m, 2H), 4.44–4.35 (m, 2H), 4.30 (m, 1H), 3.88 (m, 2H), 3.74–3.67 (m, 3H), 3.61–3.15 (m, 6H), 2.49–2.30 (m, 2H), 2.23 (m, 2H), 1.97 (m, 2H), 1.93–1.74 (m, 6H), 1.48 (s, 9H). HRMS-TOF (*m/z*) calcd for C_32_H_41_FN_8_O_4_ [M + H]^+^: 621.3308, found 621.3405.

#### 4.1.16. tert-Butyl 4-(2-((endo)-3-((8-(4-cyano-2-fluorophenyl)-7,8-dihydro-6H-pyrimido[5,4-b][1,4]oxazin-4-yl)amino)-8-azabicyclo[3.2.1]octan-8-yl)acetyl)piperazine-1-carboxylate (20)

Yellowish solid, 68% yield. ^1^H-NMR (600 MHz, CDCl_3_) δ (ppm): 7.92 (s, 1H), 7.55–7.50 (m, 1H), 7.47 (m, 2H), 5.34 (d, *J* = 7.1 Hz, 1H), 4.44–4.35 (m, 2H), 4.26 (q, *J* = 6.7 Hz, 1H), 3.91–3.84 (m, 2H), 3.72–3.67 (m, 2H), 3.60 (s, 2H), 3.53–3.47 (m, 2H), 3.43 (m, 2H), 3.32 (m, 2H), 3.28 (m, 2H), 2.29 (m, 2H), 2.21–2.13 (m, 2H), 1.96 (m, 2H), 1.83 (d, *J* = 14.3 Hz, 2H), 1.50 (s, 9H). 13C-NMR (150 MHz, CDCl_3_) δ (ppm): 168.8, 156.4 (d, *J* = 250.6 Hz), 154.6, 151.3, 149.8, 143.8, 135.5 (d, *J* = 10.5 Hz), 128.5 (d, *J* = 3.5 Hz), 128.4 (d, *J* = 2.5 Hz), 121.8, 120.6 (d, *J* = 23.7 Hz), 117.6 (d, *J* = 2.5 Hz), 109.4 (d, *J* = 9.1 Hz), 80.3, 70.6, 64.4, 58.8, 56.0 (x2), 48.2 (d, *J* = 3.8 Hz), 45.8, 43.5 (x2), 42.4, 41.7, 36.9 (x2), 28.4 (x3), 26.2. HRMS-TOF (*m/z*) calcd for C_31_H_39_FN_8_O_4_ [M + H]^+^: 607.3151, found 607.3239.

#### 4.1.17. 3-Fluoro-4-(4-(piperidin-4-ylamino)-6,7-dihydro-8H-pyrimido[5,4-b][1,4]oxazin-8-yl)benzonitrile (21)

Follow the similar procedure of **14**. Yellow solid, 72% yield. MS-ESI: [M + H]^+^: 355.7.

#### 4.1.18. Isopropyl 4-((8-(4-cyano-2-fluorophenyl)-7,8-dihydro-6H-pyrimido[5,4-b][1,4]oxazin-4-yl)amino)piperidine-1-carboxylate (22)

Follow the similar procedure of **15**. Yellowish solid, 70% yield. ^1^H-NMR (600 MHz, CDCl_3_) δ (ppm): 7.93 (s, 1H), 7.56–7.51 (m, 1H), 7.48 (m, 2H), 4.95 (dt, *J* = 12.5, 6.2 Hz, 1H), 4.83 (d, *J* = 8.1 Hz, 1H), 4.43–4.33 (m, 2H), 4.27–4.08 (m, 3H), 3.93–3.85 (m, 2H), 3.00 (t, *J* = 12.0 Hz, 2H), 2.13–2.04 (m, 2H), 1.50–1.37 (m, 2H), 1.28 (s, 3H), 1.27 (s, 3H). 13C-NMR (150 MHz, CDCl_3_) δ (ppm): 156.4 (d, *J* = 250.6 Hz), 155.2, 151.2, 149.8, 144.2, 135.4 (d, *J* = 10.4 Hz), 128.5 (d, *J* = 3.8 Hz), 128.4 (d, *J* = 2.6 Hz), 121.7, 120.6 (d, *J* = 23.7 Hz), 117.6 (d, *J* = 2.7 Hz), 109.5 (d, *J* = 9.2 Hz), 68.7, 64.3, 48.2 (d, *J* = 3.9 Hz), 47.6, 42.8 (x2), 32.5 (x2), 22.3 (x2). HRMS-TOF (*m/z*) calcd for C_22_H_25_FN_6_O_3_ [M + H]^+^: 441.2045, found 441.2144.

#### 4.1.19. 4-(4-((1-(5-Ethylpyrimidin-2-yl)piperidin-4-yl)amino)-6,7-dihydro-8H-pyrimido[5,4-b][1,4]oxazin-8-yl)-3-fluorobenzonitrile (23)

Follow the similar procedure of **16**–**20**. Yellowish solid, 54% yield. ^1^H-NMR (600 MHz, CDCl_3_) δ (ppm): 8.20 (s, 2H), 7.95 (s, 1H), 7.56–7.51 (m, 1H), 7.48 (m, 2H), 4.87 (d, *J* = 8.2 Hz, 1H), 4.68 (m, 2H), 4.42–4.32 (m, 2H), 4.32–4.17 (m, 1H), 3.92–3.84 (m, 2H), 3.24–3.10 (m, 2H), 2.49 (q, *J* = 7.6 Hz, 2H), 2.21–2.12 (m, 2H), 1.50 (m, 2H), 1.21 (t, *J* = 7.6 Hz, 3H). 13C-NMR (150 MHz, CDCl_3_) δ (ppm): 160.8, 157.2 (x2), 156.4 (d, *J* = 250.9 Hz), 151.4, 149.8, 144.1, 135.4 (d, *J* = 10.5 Hz), 128.5 (d, *J* = 3.7 Hz), 128.4 (d, *J* = 2.6 Hz), 124.5, 121.7, 120.6 (d, *J* = 23.8 Hz), 117.7 (d, *J* = 2.6 Hz), 109.4 (d, *J* = 9.0 Hz), 64.3, 48.2 (d, *J* = 3.9 Hz), 48.0, 43.1 (x2), 32.5 (x2), 22.7, 15.7. HRMS-TOF (*m/z*) calcd for C_24_H_25_FN_8_O [M + H]^+^: 461.2208, found 461.2318.

#### 4.1.20. 4-(4-((1-(5-Chloropyrimidin-2-yl)piperidin-4-yl)amino)-6,7-dihydro-8H-pyrimido[5,4-b][1,4]oxazin-8-yl)-3-fluorobenzonitrile (24)

Follow the similar procedure of **16**–**20**. Yellowish solid, 58% yield. ^1^H-NMR (600 MHz, CDCl_3_) δ (ppm): 8.25 (s, 2H), 7.95 (s, 1H), 7.58–7.51 (m, 1H), 7.48 (m, 2H), 4.86 (d, *J* = 8.1 Hz, 1H), 4.74–4.61 (m, 2H), 4.47–4.33 (m, 2H), 4.28 (m, 1H), 3.96–3.79 (m, 2H), 3.27–3.11 (m, 2H), 2.21–2.12 (m, 2H), 1.48 (m, 2H). 13C-NMR (150 MHz, CDCl_3_) δ (ppm): 159.8, 156.4 (d, *J* = 250.9 Hz), 155.9 (x2), 151.3, 149.8, 144.2, 135.4 (d, *J* = 10.4 Hz), 128.5 (d, *J* = 3.6 Hz), 128.4 (d, *J* = 2.5 Hz), 121.7, 120.6 (d, *J* = 23.7 Hz), 118.0, 117.6, 109.4 (d, *J* = 9.2 Hz), 64.3, 48.2 (d, *J* = 3.8 Hz), 47.8, 43.2 (x2), 32.8 (x2). HRMS-TOF (*m/z*) calcd for C_22_H_20_ClFN_8_O [M + H]^+^: 467.1505, found 467.1613.

#### 4.1.21. tert-Butyl 4-(2-(4-((8-(4-cyano-2-fluorophenyl)-7,8-dihydro-6H-pyrimido[5,4-b][1,4]oxazin-4-yl)amino)piperidin-1-yl)acetyl)piperazine-1-carboxylate (25)

Follow the similar procedure of **16**–**20**. Yellowish solid, 63% yield. ^1^H-NMR (600 MHz, CDCl_3_) δ (ppm): 7.91 (s, 1H), 7.54–7.49 (m, 1H), 7.46 (m, 2H), 4.84 (d, *J* = 8.1 Hz, 1H), 4.50–4.30 (m, 2H), 4.01 (m, 1H), 3.94–3.81 (m, 2H), 3.59 (s, 4H), 3.51–3.45 (m, 2H), 3.42 (s, 2H), 3.22 (s, 2H), 2.89 (m, 2H), 2.32 (m, 2H), 2.08 (m, 2H), 1.65–1.53 (m, 2H), 1.49 (s, 9H). 13C-NMR (150 MHz, CDCl_3_) δ (ppm): 168.4, 156.4 (d, *J* = 250.6 Hz), 154.7, 151.4, 149.8, 144.0, 135.5 (d, *J* = 10.5 Hz), 128.5 (d, *J* = 3.6 Hz), 128.4 (d, *J* = 2.5 Hz), 121.7, 120.6 (d, *J* = 23.7 Hz), 117.6 (d, *J* = 2.6 Hz), 109.4 (d, *J* = 9.1 Hz), 80.3, 64.3, 61.4, 52.8, 50.8, 48.2 (d, *J* = 3.8 Hz), 47.0, 45.5, 43.6 (x2), 41.7, 32.6 (x2), 28.4 (x3). HRMS-TOF (*m/z*) calcd for C_29_H_37_FN_8_O_4_ [M + H]^+^: 581.2995, found 581.3136.

### 4.2. hGPR119 agonistic activity

CHO K1 cells stably transfected with human GPR119 were grown at 37 °C, 95%O_2_ and 5% CO_2_ in 75 cm flasks containing DMEM/F12 (1:1) media with added 10% FBS (Gibco^®^), Geneticin (Gibco^®^) and grown until 90% confluent. Cells were then washed (PBS), lifted with cell dissociation solution (Invtrogen^®^), counted and used for cAMP accumulation assays and/or passaging (1:10). Following the manufacturer’s instructions for the LANCE^®^ Ultra cAMP assay (Perkin Elmer), cell transfected with *h*GPR119 were centrifuged (1000 rpm, 5 min), re-suspended in cAMP assay buffer (HBSS, 0.1% BSA, 0.5 mM IBMX and 5 mM HEPES) and seeded at 5000 cells/well in optiplate-384 (Perkin Elmer). Cells were treated with compounds or reference **GSK1292263** over a range of concentrations (10 μM-0.6 μM) and incubated for 1 h. Cell lysis buffers (4X Eu-cAMP tracer solution and 4X ULight™-anti-cAMP solution) were added to each well, and the plates were incubated at r. t. for 1 h before being read on Envision (Perkin Elmer). The assay was performed for three replicates for each concentration.

### 4.3. oGTT in C57BL/6N mice

For the acute single dose study, vehicle (0.5% carboxymethylcellulose sodium, 10 ml/kg), compound **10**, compound **15** (15 and 30 mg/kg) and vildagliptin (30 mg/kg) were administered to C57BL/6N mice after 16-h starvation, then the oral glucose tolerance test (3 g/kg) was conducted after 4 h of the single dose, the blood glucose level at 0, 15, 30, 60, 90, and 120 min were recorded for area under curve calculation (AUC_0–2h_). AUC_0–2h_(mmol/L) = (BG_0_+BG_15_)×0.25/2 + (BG_15_+BG_30_)×0.25/2 + (BG_30_+BG_60_)×0.5/2 + (BG_60_+BG_90_)×0.5/2 + (BG_90_+BG_120_)×0.5/2.

## References

[1] Skyler JS. Diabetes mellitus: pathogenesis and treatment strategies. J Med Chem 2004;47:4113–7. 15293979 10.1021/jm0306273

[2] Nieto A, Fernández-Vega V, Spicer TP, et al. Identification of novel, structurally diverse, small molecule modulators of gpr119. Assay Drug Dev Techn 2018;16:278–88. 10.1089/adt.2018.849 PMC6065524 30019946

[3] Lan J, Zhao Y, Dong F, et al. Meta-analysis of the effect and safety of berberine in the treatment of type 2 diabetes mellitus, hyperlipemia and hypertension. J Ethnopharmacol 2015;161:69–81. 25498346 10.1016/j.jep.2014.09.049

[4] Campbell PT, Newton CC, Patel AV, et al. Diabetes and cause-specific mortality in a prospective cohort of one million U.S. adults. Diabetes Care 2012;35:1835–44. 22699290 10.2337/dc12-0002 PMC3425000

[5] Koro CE, Bowlin SJ, Bourgeois N, et al. Glycemic control from 1988 to 2000 among U.S. adults diagnosed with type 2 diabetes a preliminary report. Diabetes Care 2004;27:17–20. 14693960 10.2337/diacare.27.1.17

[6] Fang Y, Yang Z, Gundeti S, et al. Novel 5-nitropyrimidine derivatives bearing endo-azabicyclic alcohols/amines as potent GPR119 agonists. Bioorgan Med Chem 2017;25:254–60. 10.1016/j.bmc.2016.10.030 27825553

[7] Wacker DA, Wang Y, Broekema M, et al. Discovery of BMS-903452, an anti-diabetic clinical candidate targeting GPR119. Clinical and Translational Imaging 2014;57:7499. 10.1021/jm501175v 25208139

[8] Scott JS, Bowker SS, Brocklehurst KJ, et al. Circumventing seizure activity in a series of G protein coupled receptor 119 (GPR119) agonists. J Med Chem 2014;57:8984–98. 25286150 10.1021/jm5011012

[9] Yang Z, Fang Y, Park H. Synthesis and biological evaluation of pyrimidine derivatives with diverse azabicyclic ether/amine as novel GPR119 agonist. Bioorg Med Chem Lett 2017;27:2515–9. 28408218 10.1016/j.bmcl.2017.03.092

[10] Kaushik AC, Kumar A, Rehman AU, et al. Deciphering G-Protein-Coupled Receptor 119 Agonists as Promising Strategy against Type 2 Diabetes Using Systems Biology Approach. ACS Omega 2018;3:18214–26.

[11] Overton HA, Fyfe MCT, Reynet C. GPR119, a novel G protein-coupled receptor target for the treatment of type 2 diabetes and obesity. Br J Pharmacol 2009;153:S76–S81. 10.1038/sj.bjp.0707529 PMC2268073 18037923

[12] Han T, Lee BM, Park YH, et al. YH18968, a novel 1,2,4-triazolone G-protein coupled receptor 119 agonist for the treatment of type 2 diabetes mellitus. Biomol Ther 2018;26:201–9. 10.4062/biomolther.2018.011 PMC5839499 29495245

[13] Koshizawa T, Morimoto T, Watanabe G, et al. Optimization of a novel series of potent and orally bioavailable GPR119 agonists. Bioorg Med Chem Lett 2017;27:3249–53. 28648463 10.1016/j.bmcl.2017.06.034

[14] Zhou Y, Zhu X, Zhang L, et al. Design, synthesis, and biological evaluation of 2-(4-(methylsulfonyl)phenyl)pyridine derivatives as GPR119 agonists. Chem Biol Drug Des 2019;93:67–74. 30120879 10.1111/cbdd.13380

[15] Gao J, Tian L, Weng G, et al. Stimulating beta cell replication and improving islet graft function by GPR119 agonists. Transpl Int 2011;24:1124–34. 21902730 10.1111/j.1432-2277.2011.01332.x

[16] Fyfe MC, McCormack JG, Overton HA, et al. GPR119 agonists as potential new oral agents for the treatment of type 2 diabetes and obesity. Expert Opin Drug Dis 2008;3:403–13. 10.1517/17460441.3.4.403 23489096

[17] Yoshida S, Ohishi T, Matsui T, et al. The role of small molecule GPR119 agonist, AS1535907, in glucose‐stimulated insulin secretion and pancreatic β‐cell function. Diabetes Obes Metab 2011;13:34–41. 21114601 10.1111/j.1463-1326.2010.01315.x

[18] Matsuda D, Kawamura M, Kobashi Y, et al. Design, synthesis and biological evaluation of novel 7-azaspiro[3,5]nonane derivatives as GPR119 agonists. Bioorgan Med Chem 2018;26:1832–47. 10.1016/j.bmc.2018.02.032 29486951

[19] Zhu C, Wang L, Zhu Y, et al. Discovery of phenyl acetamides as potent and selective GPR119 agonists. Bioorg Med Chem Lett 2017;27:1124–8. 28185720 10.1016/j.bmcl.2017.01.091

[20] Li G, Huan Y, Yuan BK, et al. Discovery of novel xanthine compounds targeting DPP-IV and GPR119 as anti-diabetic agents. Eur J Med Chem 2016;124:103–16. 27560285 10.1016/j.ejmech.2016.08.023

[21] Jang YK, Lee KM, Jung K, et al. Design, synthesis, and biological evaluation of aryl N-methoxyamide derivatives as GPR119 agonists. Bioorg Med Chem Lett 2017;27:3909–14. 28666737 10.1016/j.bmcl.2017.06.032

[22] Matsuda D, Kobashi Y, Mikami A, et al. Novel 3 H-[1,2,3]triazolo[4,5- c]pyridine derivatives as GPR119 agonists: Synthesis and structure-activity/solubility relationships. Bioorgan Med Chem 2017;25:4339–54. 10.1016/j.bmc.2017.06.014 28662959

[23] Harada K, Mizukami J, Kadowaki S, et al. Design and synthesis of novel and potent GPR119 agonists with a spirocyclic structure. Bioorg Med Chem Lett 2018;28:1228–33. 29519733 10.1016/j.bmcl.2018.02.044

[24] Han S, Narayanan S, Sun HK, et al. Discovery of a novel trans-1,4-dioxycyclohexane GPR119 agonist series. Bioorg Med Chem Lett 2015;25:3034–8. 26048791 10.1016/j.bmcl.2015.04.102

[25] Neelamkavil SF, Stamford AW, Kowalski T, et al. Discovery of MK-8282 as a potent G-protein-coupled receptor 119 agonist for the treatment of type 2 diabetes. Acs Med Chem Lett 2018;9:457–61. 29795759 10.1021/acsmedchemlett.8b00073 PMC5949837

[26] Ritter K, Buning C, Halland N, et al. G protein-coupled receptor 119 (gpr119) agonists for the treatment of diabetes: recent progress and prevailing challenges. J Med Chem 2016;59:3579–92. 26512410 10.1021/acs.jmedchem.5b01198

[27] Wacker DA, Wang Y, Broekema M, et al. Discovery of 5-chloro-4-((1-(5-chloropyrimidin-2-yl)piperidin-4-yl)oxy)-1-(2-fluoro-4-(methylsulfonyl)phenyl)pyridin-2(1h)-one (BMS-903452), an antidiabetic clinical candidate targeting GPR119. J Med Chem 2014;57:7499–508. 25208139 10.1021/jm501175v

[28] Semple G, Fioravanti B, Pereira G, et al. Discovery of the First Potent and Orally Efficacious Agonist of the Orphan G-Protein Coupled Receptor 119. J. Med. Chem 2008;51:5172–5. 18698756 10.1021/jm8006867

[29] Liu P, Hu Z, Dubois BG, et al. Design of potent and orally active GPR119 agonists for the treatment of type ii diabetes. Acs Med Chem Lett 2015;6:936–41. 26288697 10.1021/acsmedchemlett.5b00207 PMC4538435

[30] Yang Z, Fang Y, Pham TN, et al. Synthesis and biological evaluation of 5-nitropyrimidine analogs with azabicyclic substituents as GPR119 agonists. Bioorg Med Chem Lett 2013;23:1519–21. 23374864 10.1016/j.bmcl.2012.12.011

[31] Fang Y, Jun X, Zhifeng L, et al. Design and synthesis of novel pyrimido[5,4-d]pyrimidine derivatives as GPR119 agonist for treatment of type 2 diabetes. Bioorgan Med Chem 2018;26:4080–7. 10.1016/j.bmc.2018.06.035 30100020

[32] Fang Y, Xiong L, Hu J, et al. Synthesis and evaluation of novel fused pyrimidine derivatives as GPR119 agonists. Bioorg Chem 2019;86:103–11. 30685641 10.1016/j.bioorg.2019.01.032

[33] Pham TAN, Yang Z, Fang Y, et al. Synthesis and biological evaluation of novel 2,4-disubstituted quinazoline analogues as GPR119 agonists. Bioorgan Med Chem 2013;21:1349–56. 10.1016/j.bmc.2012.12.013 23357035

[34] Heidmann B, Gatfield J, Roch C, et al. Discovery of highly potent dual orexin receptor antagonists via a scaffold-hopping approach. Chemmedchem 2016;11:2132–46. 27390287 10.1002/cmdc.201600175

[35] Ryan H; Jennifer K; Melissa F, et al. Pyrimidine compounds as JAK kinase inhibitors, Patent WO 2017189822; 2017.

[36] Mastracchio A, Bruncko M, Lai C, et al. Heterocyclylpyridine derivatives as CDK9 kinase inhibitors and their preparation, Patent US 20140275011; 2014.

